# Case Report: Marchiafava-Bignami disease: anti-parietal cell antibodies as a potential etiological factor in a novel case presentation

**DOI:** 10.3389/fmolb.2026.1787073

**Published:** 2026-03-18

**Authors:** Junhao Zhang, Lizhen Wang, Shuangfeng Yang, Yinhong Xie, Chengcheng He, Hongyu Lin, Yang Li, Shanfeng Qiu, Shunxian Wang, Shengxiong Pu, Pei Wang, Lei Zhang, Jian Chen, Yang Peng, Yun Liu, Ying Ma

**Affiliations:** 1 Department of Neurology, Affiliated Hospital of North Sichuan Medical College, Nanchong, China; 2 Department of Neurology, Yuechi County People’s Hospital, Guangan, China; 3 Department of Neurology, Guangyuan First People’s Hospital, Guangyuan, China; 4 Department of Neurology, Tongliang District People’s Hospital, Chongqing, China; 5 Department of Forensic Pathology, School of Basic Medicine and Forensic Medicine, North Sichuan Medical College, Nanchong, China

**Keywords:** anti-parietal cell antibodies, demyelination, marchiafava-Bignami disease (MBD), necrosis, vitamin B12 deficiency

## Abstract

Marchiafava-Bignami disease (MBD) represents a rare neurological disorder predominantly associated with chronic ethanol consumption and thiamine insufficiency. However, its possible correlation with cobalamin deficiency resulting from anti-parietal cell antibodies (APCA) remained inadequately characterized in the literature. Timely diagnosis and therapeutic intervention were critical for optimizing clinical outcomes. This case suggested that APCA screening and vitamin B12 assessment should be considered in the diagnostic workup of MBD, especially in patients without classic thiamine deficiency.

## 1 Introduction

Marchiafava-Bignami disease (MBD) is a rare and frequently devastating neurological disorder, pathologically characterized by selective demyelination and necrosis of the corpus callosum (Singer et al., 2023). Although its exact pathophysiology remained incompletely understood, current evidence suggested a multifactorial mechanism involving oxidative stress, mitochondrial dysfunction, ethanol-induced neurotoxicity, and concomitant thiamine deficiency due to malnutrition (Fernandes et al., 2017). Clinically, MBD exhibited a broad spectrum of manifestations, ranging from acute presentations such as encephalopathy, seizures, and coma to chronic neuropsychiatric deficits, including dementia, gait abnormalities, and frontal lobe dysfunction (Ren and Wang, 2024). Diagnosis primarily relied on MRI imaging, which played a pivotal role in identifying characteristic structural abnormalities. To date, no standardized treatment guidelines were available; however, timely intervention was critical for improving prognosis. Although the disease was once considered fatal, advancements in imaging and early thiamine supplementation have improved outcomes, with some patients achieving partial or complete recovery (Sidhpura et al., 2024). Nevertheless, prognosis remained highly variable, and was influenced by factors such as disease stage, the extent of radiological lesions, and the prompt initiation of treatment.

Against this classical framework, the present study reported a clinical case of MBD in a patient with concurrent positive anti-parietal cell antibodies (APCA), manifesting as low serum vitamin B12 (118 pg/mL; reference range 174–878 pg/mL) and normal vitamin B1 (1.47 ng/mL; reference range 0.22–2.65 ng/mL) levels prior to any supplementation—an atypical etiological profile that deviated from MBD’s traditional association with thiamine deficiency—and proceeded to discuss the underlying pathophysiological mechanisms, clinical presentations, neuroimaging characteristics, treatment strategies, and prognostic implications specific to this form of MBD.

## 2 Case description

A 45-year-old male patient was admitted due to progressive cognitive decline, specifically characterized by memory deterioration and psychomotor retardation over the past 2 months without any identifiable precipitating factors. The patient exhibited bradykinesia and mild dysarthria. No accompanying symptoms such as fever, chills, vertigo, seizures, or episodes of sudden falls were reported. Pre-admission MRI conducted from the referring hospital revealed symmetrical, patchy hyperintense lesions within the bilateral corpus callosum and pontocerebellar peduncles areas. There was no notable past medical history aside from a longstanding history of chronic alcohol consumption spanning over 2 decades, with a daily intake of 100 mL of high-alcohol liquor. The patient had no vegetarian history or food deprivation, and anthropometric indicators were unremarkable. The vital signs were stable. Neurological examination showed an alert and responsive individual with evident dysarthria. Cognitive assessment revealed deficits in comprehension, recent memory, calculation, and orientation. The remainder of the physical examination was unremarkable.

**FIGURE 1:**
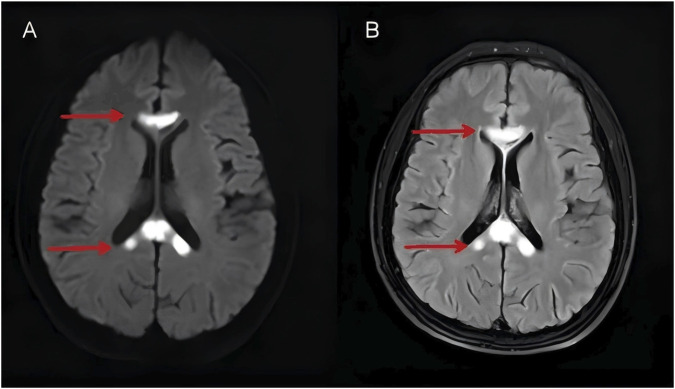
Axial DWI **(A)** and T2-FLAIR **(B)** images on admission demonstrated symmetrical hyperintense lesions with restricted diffusion (arrows) in the genu and splenium of the corpus callosum, consistent with acute demyelination.

Comprehensive laboratory investigations revealed the following significant findings (Table 1). Notably, serum vitamin B1 level was normal (1.47 ng/mL; reference range 0.22–2.65 ng/mL, based on serum sampled prior to any supplementation), in contrast to low serum vitamin B12 levels (118 pg/mL; reference range 174–878 pg/mL, based on serum sampled prior to any supplementation)—a key finding—and positive anti-parietal cell antibody IgG (APCA-IgG), which were the most clinically relevant results. Complete blood count (CBC) revealed a slightly elevated absolute neutrophil count (8.07 × 10^9^/L), a reduced red blood cell (RBC) count (3.84 × 10^12^/L), and an increased mean corpuscular volume (MCV) (102.30 fL), consistent with the aforementioned vitamin B12 deficiency-related anemia. Gamma-glutamyl transferase (GGT) (a hepatic function marker) was elevated (90 U/L). Laboratory investigations yielded two critical findings: anti-intrinsic factor antibody was negative and homocysteine level was within the normal range. All other measured parameters—including renal and hepatic function, serum lipids, glucose, electrolytes, cardiac biomarkers, coagulation profile, connective tissue antibodies, thyroid indices, diurnal ACTH/cortisol levels, viral serologies, and routine urinalysis—were within their respective reference ranges or negative. Routine and biochemical cerebrospinal fluid (CSF) tests showed no significant abnormalities; additionally, autoimmune encephalitis and central nervous system demyelinating diseases yielded negative or within-reference-range findings. Specifically, IgG antibodies targeting NMDAR, AMPA1, AMPA2, LGI1, CASPR2, and GABABR receptors were absent in both cerebrospinal fluid and serum specimens. Cerebrospinal fluid oligoclonal band testing demonstrated a type IV (negative) pattern, and corresponding intrathecal synthesis metrics—including the IgG index and 24-h intrathecal synthesis rate—remained within physiological parameters. Collectively, these laboratory data do not demonstrate the characteristic immunological profile observed in disorders such as multiple sclerosis, although these etiologies cannot be entirely excluded. Methylmalonic acid testing was not performed due to laboratory limitations. Subsequently, the Mini-Mental State Examination (MMSE) yielded a score of 12, and the Montreal Cognitive Assessment (MoCA) a score of 5, indicating moderate cognitive impairment in the patient. Three-dimensional video electroencephalogram (EEG) monitoring revealed mild diffuse slowing. Diffusion-weighted imaging (DWI) (Figure 1A) demonstrated areas of significantly restricted water molecule diffusion in the affected corpus callosum, consistent with acute cytotoxic edema and demyelination. Corresponding T2-FLAIR sequences (Figure 1B) revealed symmetrical hyperintense lesions involving the genu, body, and splenium of the corpus callosum, as well as the bilateral pontocerebellar regions. Notably, these lesions demonstrated no significant enhancement on post-contrast imaging. Magnetic resonance angiography (MRA) identified a small saccular aneurysm arising from the left middle cerebral artery; additionally, there was congenital aplasia of the A1 segment of the right anterior cerebral artery, with no other significant cerebrovascular anomalies observed. Moreover, the direct endoscopic evaluation of the gastric mucosa was not performed due to patient refusal.

**TABLE 1:** Key laboratory findings.

Test item	Specimen type	Result	Unit	Reference range
Vitamin B1	Serum	1.47	ng/mL	0.22–2.65
Vitamin B12	Serum	118	pg/mL	174–878
Anti-parietal cell antibody (APCA)-IgG	Serum	29.26	U/mL	0–20
Anti-intrinsic factor antibody (Anti-IF)	Serum	<0.5	U/mL	0–20
Homocysteine (HCY)	Plasma	14.8	µmol/L	1.0–15.0
Autoimmune encephalitis antibodies^a^	Peripheral blood and cerebrospinal fluid	Negative		
Oligoclonal band (OCB)	Serum and cerebrospinal fluid	Type IV^b^ (Negative)		
Cerebrospinal fluid (CSF) IgG index	Serum and cerebrospinal fluid	0.5	—	<0.70
24-h intrathecal synthesis rate		−1.6	mg/24h	−9.9–3.3 mg/24 h
Red blood cell count (RBC)	Whole blood	3.84	×10^12^/L	4.3–5.8 (male) 3.8–5.1 (female)
Mean corpuscular volume (MCV)	Whole blood	102.30	fL	82–100
Absolute Neutrophil count	Whole blood	8.07	×10^9^/L	2.0–7.0
Gamma-glutamyl transferase (GGT)	Plasma	90	U/L	10–60

^a^ Panel includes NMDAR, AMPA1, AMPA2, LGI1, CASPR2, and GABABR, antibodies.

^b^ Oligoclonal bands were detected in both serum and cerebrospinal fluid, with identical banding patterns in each, not indicative of the typical intrathecal synthesis pattern seen in multiple sclerosis.

**FIGURE 2:**
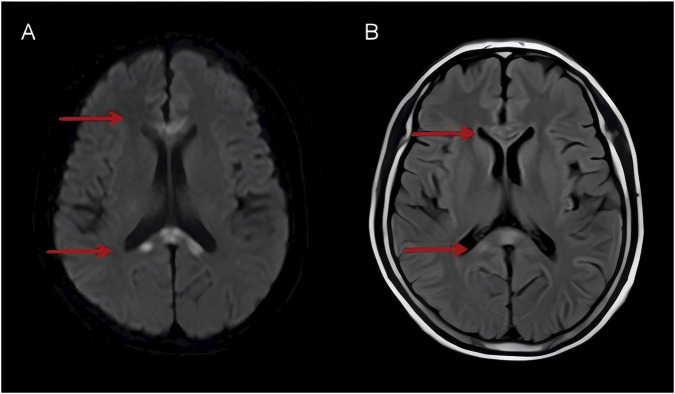
T2-FLAIR **(A)** and DWI **(B)** demonstrated improvement of the abnormal signals on DWI and FLAIR compared to prior images.

Based on the patient’s clinical history, neurological presentation, and the accessory examination, MBD was diagnosed. Following admission, a series of laboratory and auxiliary examinations were completed, upon which treatment was immediately initiated with intramuscular injections of thiamine (100 mg twice daily) and methylcobalamin (500 μg once daily). In parallel, low-dose corticosteroids (dexamethasone sodium phosphate injection, 10 mg) were administered by intravenous infusion, and neurotrophic agents were prescribed. By the sixth day of hospitalization, the patient’s neurological symptoms had significantly improved, meeting criteria for discharge. Post-discharge plan included continued intramuscular supplementation of both vitamin B1 and methylcobalamin. An oral corticosteroid tapering protocol was prescribed (prednisone acetate tablets, starting at 45 mg daily, reduced to 30 mg daily after 1 week, and then tapered by 10 mg per week until discontinuation), along with potassium and calcium supplementation and gastric mucosal protection (Table 2). A follow-up brain MRI was scheduled 2 weeks after discharge. Compared to the prior imaging findings, the abnormal signals observed in both T2-FLAIR (Figure 2A) and DWI (Figure 2B) demonstrated significant attenuation, with notable improvement also noted on cognitive assessment (MMSE 18; MoCA 10).

**TABLE 2:** Chronological timeline of the patient’s diagnostic assessments, therapeutic interventions, and clinical progression.

Time point	Examinations	Laboratory tests	Clinical scores	Treatment
2024.12.24 (admission)	1. Brain MRI (unenhanced and contrast-enhanced), MRS, and MRA 2. Neuropsychological assessments: Mini-mental state examination (MMSE), Montreal Cognitive assessment (MoCA) 3. Three-dimensional video-EEG monitoring 4. Transthoracic echocardiography (TTE), abdominal ultrasonography, electrocardiogram (ECG)	1. Basic panels: Complete blood count, urinalysis, stool analysis + occult blood test, liver and renal function, electrolytes, lipid profile, cardiac injury markers, thyroid function, pre-transfusion screening 2. Immunological tests: Anti-neutrophil cytoplasmic antibodies (ANCA) + anti-glomerular basement membrane antibodies (anti-GBM), connective tissue disease antibody screen, anticardiolipin antibodies, humoral immunity (IgE, IgG, IgA, IgM, C3, C4), anti-thyroglobulin (TgAb), anti-thyroid peroxidase (TPOAb), herpes simplex virus antibodies 3. Others: Anemia workup (ferritin, vitamin B12, folate, erythropoietin), homocysteine level, cortisol and adrenocorticotropic hormone (ACTH) levels (sampled at 00:00, 08:00, 16:00), epstein-barr virus DNA, cytomegalovirus DNA.	MMSE: 12 MoCA: 5	—
2024.12.25	1. Completion of pending examinations 2. Lumbar puncture	1. Cerebrospinal fluid (CSF): routine, biochemistry, and bacterial culture 2. External laboratory tests Anti-intrinsic factor antibody (IFA) and IgG anti-parietal cell antibody (APCA) Serum vitamin B1 level Paired serum and CSF oligoclonal band (OCB) analysis with quantitative indices (e.g., IgG index, 24-h intrathecal synthesis rate) Autoimmune encephalitis antibody panel in serum and CSF (anti-NMDAR, -AMPA1, -AMPA2, -LGI1, -CASPR2, -GABABR IgG)	—	Post-lumbar puncture intravenous hydration
2024.12.26	—	—	—	1. Intramuscular Injection: Thiamine (100 mg twice daily) and methylcobalamin (500 μg once daily) 2. Neurotrophic agent: Monosialotetrahexosylganglioside sodium (60 mg intravenous infusion daily) 3. Low-dose corticosteroid Dexamethasone sodium phosphate (10 mg intravenous infusion daily)
2024.12.27–2024.12.30	—	—	—	Treatment regimen identical to that administered on 2024.12.26
Follow-up (approx. 2 weeks post-discharge)				
2025.01.16	1. Unenhanced brain MRI 2. Neuropsychological assessments: Mini-mental state examination (MMSE), Montreal Cognitive assessment (MoCA)	—	MMSE: 1 MoCA: 10	Discharge plan: Continue intramuscular supplementation of both vitamin B1 and methylcobalamin. A tapering regimen of oral corticosteroids was prescribed (prednisone acetate tablets, starting at 45 mg daily, reduced to 30 mg daily after 1 week, and then tapered by 10 mg per week until discontinuation), along with potassium/calcium supplements and gastroprotective agents
	Imaging findings (2025.01.16): Follow-up brain MRI showed marked improvement. Multiple patchy abnormal signals were noted within the corpus callosum, appearing hypointense on T1-weighted imaging (T1WI) and hyperintense on T2-weighted (T2WI) and T2-FLAIR sequences, with restricted diffusion on DWI. These findings demonstrated significant resolution compared to the initial MRI (2024.12.24)			
Clinical course	Scheduled follow-up assessments at 1 and 3 months post-discharge were discontinued at the patient’s request, as they reported near-complete symptomatic recovery and declined further testing			

## 3 Discussion

**FIGURE 3:**
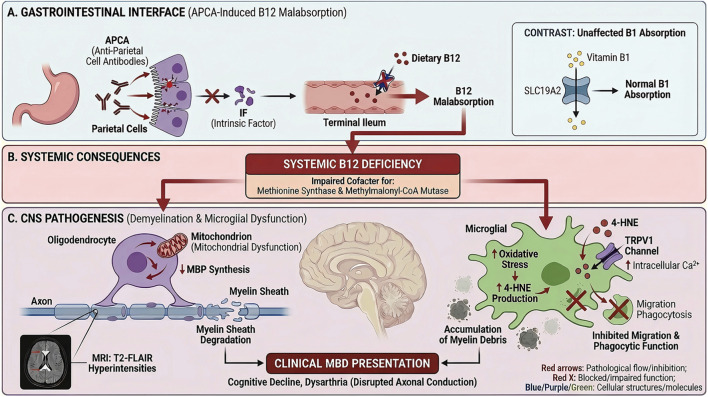
Pathological Mechanism of APCA-Induced Vitamin B12 Deficiency Potentially Contributing to Demyelination in Atypical Marchiafava-Bignami Disease. The diagram illustrates two potential pathogenic mechanisms contributing to demyelination, both stemming from APCA-mediated Vitamin B12 deficiency. The initial etiology involves APCA-mediated destruction of parietal cells and reduced Intrinsic Factor (IF) secretion, causing selective Vitamin B12 malabsorption. The resulting deficiency acted as a precipitating trigger by impairing two key enzyme systems critical for oligodendrocyte function: methionine synthase and methylmalonyl-CoA mutase. This systemic Vitamin B12 deficiency is proposed to drive CNS pathology through two distinct pathways: (Left Panel) Deficiency impairs oligodendrocyte mitochondrial function and reduces Myelin Basic Protein (MBP) synthesis, leading to myelin sheath degradation. (Right Panel) Deficiency elevates microglial oxidative stress and promotes 4-hydroxynonenal (4-HNE) production, which activates TRPV1 channels and inhibits microglial migration and phagocytosis. Both mechanisms are presented as potential causes contributing to the accumulation of myelin debris and the development of MBD lesions.

MBD was first identified in 1903 by Italian pathologists Ettore Marchiafava and Amico Bignami during autopsies of individuals who had experienced seizures and coma following excessive consumption of low-quality red wine. It was initially described as a rare condition characterized by necrosis and demyelination of the corpus callosum (Izquierdo et al., 1992). MBD was most commonly associated with chronic alcoholism and malnutrition, particularly in individuals with long-term alcohol abuse, frequently exhibiting concurrent alcohol-related conditions such as Wernicke’s encephalopathy (Serdaru et al., 1988). However, non-alcoholic cases had also been reported, especially in patients with malnutrition secondary to gastric bypass surgery (Sidhpura et al., 2024). Although the disease most commonly affected middle-aged men between 30 and 60 years old, cases had been documented in both younger (27 years) and older (72 years) individuals (Kinsley et al., 2020; Menrai et al., 2024). Emerging evidence indicated that MBD lesions predominantly involved acute or subacute demyelination within the corpus callosum. The hemispheric white matter, cerebellar peduncles, and basal ganglia might also be involved (Waack et al., 2023). The pathophysiological mechanisms underlying MBD remained incompletely understood, Ethanol-induced neurotoxicity, metabolic disturbances, particularly thiamine deficiency, and vascular injury had been implicated in disease pathogenesis. Furthermore, oxidative stress and glial dysfunction, such as reactive astrogliosis, may exacerbate demyelination (Gramaglia et al., 2016; Ma, 2001). Notably, our case presented a critical etiological conundrum that enriched the understanding of MBD heterogeneity: the patient had a documented long-term alcohol consumption history (a classic risk factor for MBD) but also exhibited APCA-IgG positivity and isolated vitamin B12 deficiency with normal thiamine levels. This raised a key question: did alcohol merely act as a background factor, or was there a synergistic effect? We speculate that a “two-Hit” process might contribute to the observed heterogeneity, and we outline a corresponding speculative model for discussion and future testing (Figure 3). This distinction was particularly relevant given that autoimmune-mediated nutrient malabsorption has rarely been linked to MBD pathogenesis. Within our search scope of the PubMed database over the past 2 decades, no formally documented instances of MBD co-occurring with IgG-positive parietal cell antibodies were identified.

In this patient, the clinical profile was notably distinct, highlighted by three mutually supportive and lab-validated findings: to begin with, the patient tested positive for serum APCA-IgG, while other autoimmune markers typically seen in autoimmune encephalopathy were negative, excluding co-existing autoimmune conditions. Furthermore, the patient exhibited isolated vitamin B12 deficiency (118 pg/mL), aligning with APCA-mediated malabsorption, While serum thiamine levels were normal, the unreliability of serum levels as a marker of central nervous system thiamine status means that thiamine deficiency, is not precluded as a contributing factor. Lastly, neuroimaging results strongly correlated with clinical symptoms: MRI showed T2-FLAIR hyperintensities in the corpus callosum (genu, body, splenium) and pontocerebellar regions, with restricted diffusion on diffusion-weighted imaging (Figure 1). After a focused 4-week regimen of vitamin B12 (methylcobalamin) replacement therapy, combined with adjunctive thiamine supplementation, the patient exhibited partial improvement in both imaging and clinical parameters, further supporting a pathogenic link between cobalamin deficiency and lesion formation. Importantly, thiamine was administered as part of comprehensive metabolic support in MBD, despite initial normal serum levels. However, given that serum thiamine does not reliably reflect central nervous system status, a potential subtle benefit of this adjunctive therapy in the clinical course cannot be entirely excluded. In conclusion, APCA-induced vitamin B12 deficiency was highly probable—and previously underappreciated—as a potential key contributing factor to MBD in this case.

First, the first potential contributing factor appeared to be APCA-induced vitamin B12 malabsorption. The core pathological chain centered on APCA targeting parietal cell antigens, leading to parietal cell destruction and reduced intrinsic factor (IF) secretion (Venkatramanan et al., 2016; Beltramo et al., 2018). Our patient had no history of gastric surgery or dietary B12 insufficiency, suggesting that APCA may be a key factor contributing to his deficiency. Crucially, the normalization of serum thiamine levels despite long-term alcohol use suggested that the classic “alcohol-thiamine-MBD” axis cannot be definitively excluded as a possible contributor. Unlike B12 absorption, thiamine absorption via transporters like Solute Carrier Family 19 Member 2 (SLC19A2) was generally unaffected by parietal cell dysfunction (Li et al., 2024), explaining the selective nutrient deficiency. Nevertheless, we cannot dismiss the “background” contribution of chronic alcohol use, which likely served as a predisposing factor. While alcohol alone was insufficient to trigger MBD in this patient (as evidenced by the delayed onset relative to his drinking history), its chronic neurotoxic effects may have primed the central nervous system for injury. Chronic ethanol exposure was known to increase blood-brain barrier permeability and induce baseline oxidative stress in oligodendrocytes (Togre et al., 2025). It was plausible that this alcohol-induced vulnerability lowered the threshold for demyelination, rendering the corpus callosum hypersensitive to the metabolic disturbance precipitated by superimposed B12 deficiency. B12 deficiency then acted as the precipitating trigger through two interconnected processes, extrapolated from experimental models (Roth and Mohamadzadeh, 2021; Rzepka et al., 2020; Sun et al., 2023): on one hand, it impaired oligodendrocyte mitochondrial function and reduced myelin basic protein (MBP) synthesis, directly causing myelin sheath degradation that aligned with our patient’s MRI findings of T2-FLAIR hyperintensities in the corpus callosum; on the other hand, B12 deficiency increased oxidative stress in microglia, inhibiting their migration and phagocytic function. This metabolic disruption, superposed on an alcohol-compromised neural network, likely accelerated the demyelination process.

Regarding the comprehensive management of this atypical MBD case, in addition to targeted methylcobalamin replacement (addressing APCA-induced B12 deficiency) and adjunctive thiamine supplementation (mitigating potential alcohol-related metabolic disturbances), a low-dose corticosteroid regimen was also incorporated. The rationale for incorporating corticosteroids was primarily based on the patient’s neuroimaging and clinical characteristics: MRI revealed prominent demyelinating lesions in the corpus callosum and pontocerebellar regions, which are often associated with localized inflammatory responses and vasogenic edema—pathological processes that corticosteroids are well-recognized to modulate. Consistent with observations from some researchers, corticosteroids may exert beneficial effects in such scenarios by suppressing inflammatory cell infiltration, reducing blood-brain barrier permeability, and alleviating vasogenic edema, thereby relieving compressive effects on neural fibers and potentially accelerating the resolution of acute neurological symptoms (Waack et al., 2023). However, it is critical to acknowledge the ongoing debate surrounding corticosteroid use in MBD.

Therefore, the interaction between long-term alcohol use and APCA-positive B12 deficiency in this case should be viewed as synergistic rather than coincidental. Current clinical guidelines for MBD diagnosis prioritize thiamine levels and alcohol history as core diagnostic factors (Ren and Wang, 2024; Kinsley et al., 2020). However, our findings suggested that MBD etiologies were more heterogeneous than previously recognized. In clinical practice, particularly when evaluating patients with MRI findings suggestive of MBD but with normal thiamine levels, incorporating APCA and B12 screening was crucial. Recognizing this “autoimmune-metabolic” subtype allowed for precise therapeutic intervention (B12 replacement) rather than solely relying on empirical thiamine and alcohol cessation protocols. This case highlighted that MBD should be conceptualized as a heterogeneous syndrome, wherein classic risk factors such as chronic alcohol consumption may synergize with distinct metabolic insults to modulate the clinical phenotype.

## 4 Conclusion

This case underscores that APCA-mediated vitamin B12 deficiency may constitute an atypical etiological pathway for MBD, even in patients with a background of chronic alcohol consumption and preserved serum thiamine levels. The findings highlight the diagnostic importance of incorporating APCA and vitamin B12 screening in the workup of atypical MBD presentations, suggesting that chronic alcohol use may act as a synergistic predisposing factor rather than the sole causative agent in such cases.

## Data Availability

The original contributions presented in the study are included in the article/supplementary material, further inquiries can be directed to the corresponding authors.
